# Calcium-Sensing Receptor: A Key Target for Extracellular Calcium Signaling in Neurons

**DOI:** 10.3389/fphys.2016.00116

**Published:** 2016-03-30

**Authors:** Brian L. Jones, Stephen M. Smith

**Affiliations:** ^1^Division of Pulmonary and Critical Care Medicine, Department of Medicine, Oregon Health & Science UniversityPortland, OR, USA; ^2^Section of Pulmonary and Critical Care Medicine, VA Portland Health Care SystemPortland, OR, USA

**Keywords:** calcium sensing receptor, nervous system, synaptic transmission, action potentials, ion channels, calcium, excitability

## Abstract

Though both clinicians and scientists have long recognized the influence of extracellular calcium on the function of muscle and nervous tissue, recent insights reveal that the mechanisms allowing changes in extracellular calcium to alter cellular excitability have been incompletely understood. For many years the effects of calcium on neuronal signaling were explained only in terms of calcium entry through voltage-gated calcium channels and biophysical charge screening. More recently however, it has been recognized that the calcium-sensing receptor is prevalent in the nervous system and regulates synaptic transmission and neuronal activity via multiple signaling pathways. Here we review the multiplicity of mechanisms by which changes in extracellular calcium alter neuronal signaling and propose that multiple mechanisms are required to describe the full range of experimental observations.

## Calcium and excitable tissues

The importance of extracellular calcium in regulating the behavior of excitable tissues was first recognized by Sydney Ringer when he became aware that a very effective physiological saline he developed was contaminated with calcium (Ringer, 1883). Upon this discovery, Ringer quickly determined that calcium at a concentration of approximately 1 mM was essential to maintain the viability and function of isolated frog hearts and solutions derived from Ringer's work have been employed by physiologists studying the heart and many other organ systems ever since (Miller, 2004). It was some time before the importance of calcium on neuronal excitability was recognized, but despite more than 100 years of inquiry, the mechanisms by which calcium alters the excitability of neurons remain incompletely elucidated. In this mini-review we will examine some of the mechanisms by which extracellular calcium influences neuronal signaling by altering both intrinsic excitability and synaptic transmission.

## Extracellular calcium in the brain is dynamic

The distribution of calcium in the brain is characterized by steep transmembrane electrochemical gradients that are transiently attenuated as a result of large activity-dependent changes in both the intracellular and extracellular calcium concentration. The blood brain barrier defends extracellular calcium in the brain from changes in serum calcium (Jones and Keep, 1988) and at rest brain extracellular calcium is maintained at 1.1 mM (Hansen, 1985; Zhang et al., 1990; Nilsson et al., 1993, 1996). At the same time, neuronal intracellular calcium is orders of magnitude lower ranging between 50 and 100 nM, but rising rapidly to 10–100 μM in microdomains near open voltage-gated calcium channels when action potentials invade presynaptic terminals (Zucker, 1996). In contrast, during neuronal activity extracellular calcium can fall sharply as calcium is displaced to the intracellular compartment. These transient drops in calcium are facilitated by the small extracellular volume of the brain (only 12–20% of total volume; Rusakov et al., 1998) and restricted diffusion (up to five-fold slower than in free solution; Kullmann et al., 1999). The small volume and limited accessibility of the synaptic cleft led to the prediction that pre- and postsynaptic calcium influx during neurotransmission will significantly reduce calcium in the cleft following an action potential, possibly to as low as 0.3 mM (Smith, 1992; Vassilev et al., 1997b; Egelman and Montague, 1998, 1999; Rusakov et al., 1998). At the surface of the brain, ion-selective electrodes have shown that extracellular calciumfalls to 0.8 mM for tens of seconds following focal stimulation at rates of 20 Hz (Nicholson et al., 1978) and decreases to 0.1 mM have been recorded as a result of focal brain trauma (Nilsson et al., 1996). Importantly, it has been shown that short trains of action potentials can reduce extracellular calcium and impact synaptic transmission (Rusakov and Fine, 2003). The observed fall in extracellular calcium in the cortex at times of high activity (Nicholson et al., 1978) along with the extreme sensitivity of synaptic mechanism to extracellular calcium (Dodge and Rahamimoff, 1967) prompts us to ask how neurons respond to this change.

## Extracellular calcium and the intrinsic excitability of neurons

Even before calcium was identified as the trigger for exocytosis, reductions in extracellular calcium were known to increase the likelihood of action potential initiation (Frankenhaeuser and Hodgkin, 1957) by altering the properties of the neuronal ion channels. These effects on ion-channel activity can be divided into two major categories: those mediated by direct activity of calcium on ion-channel biophysics and those mediated indirectly by second messenger systems that are coupled to extracellular calcium concentrations by specific receptors. While many types of ion channels are involved in fine tuning neuronal excitability, voltage-gated sodium channels are central. Early work showed changes in excitability were mediated by shifts in the activation properties of the sodium conductance (Frankenhaeuser and Hodgkin, 1957) though subsequently extracellular calcium was found to alter the activity of other types of ion channels (Hablitz et al., 1986; Immke and McCleskey, 2001; Ma et al., 2012).

### Direct actions of calcium on neuronal excitability

**Figure 1:**
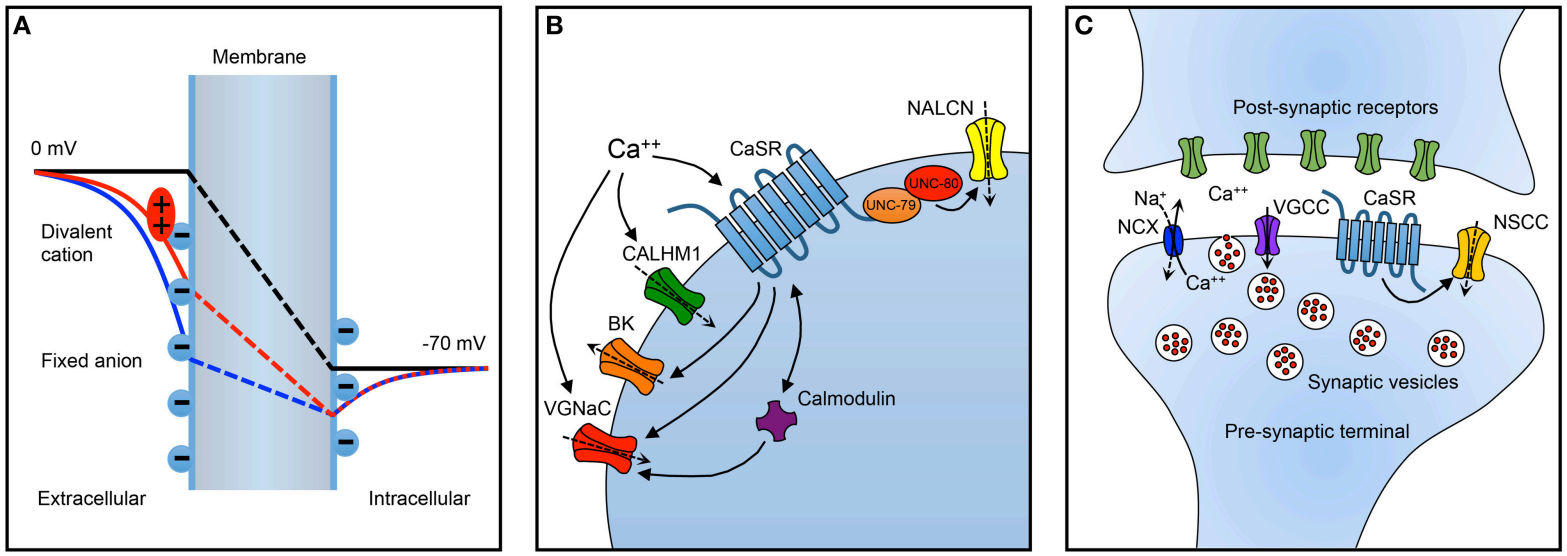
**Summary of important neuronal targets of extracellular calcium. (A)** Impact on resting membrane electric field of surface charge screening. The transmembrane potential is illustrated in three scenarios: without fixed anions (black line), with fixed anions (blue line), and with fixed anions and divalent cations (red line). The electric field produced by the transmembrane electrochemical gradient alone (black line) is attenuated by membrane associated negative charges in the absence of divalent cations (blue line). When divalent cations interact with (screen/adsorp to) the fixed anions, the influence of the fixed charges on intramembranous electric field is reduced (red line). Consequently, the activity of ion channels within the membrane and sensitive to the electric field may be altered by changes in the concentration of divalent cations. **(B)** Summary of targets of extracellular calcium on neuronal excitability and **(C)** synaptic transmission. BK, calcium-activated potassium channel; CALHM1, calcium homeostasis modulator 1; CaSR, calcium-sensing receptor; NALCN, Na-leak channel non-selective; NCX, sodium/calcium exchanger; NSCC, non-specific cation channel; VGCC, voltage-gated calcium channel; VGNaC, voltage-gated sodium channel. Dashed arrows reflect direction of current under typical conditions. Inwards arrows are depolarizing/excitatory. During high activity levels, the NCX replenishes extracellular calcium in the synaptic cleft.

The most widely recognized model for the impact of calcium on sodium channel activity is surface charge screening (aka, surface potential theory), whereby interactions between multivalent cations like calcium and negatively charged phospholipids in the neuronal membrane serve to alter the intramembrane electric field that regulates the activity of voltage-gated ion channels in a concentration dependent manner (Hille, 2001). The idea is that membrane bound negative charges influence the local potential (Figure 1A, solid blue curve) and thereby reduce the intramembranous electric field (broken blue line) established by the transmembrane electrochemical gradient (black broken line). Extracellular divalents are adsorbed to the membranous negative charges and attenuate their impact on the existing voltage field (red broken line). Hence voltage-dependent channels within the intramembranous electric field have altered activity. In the case of a high extracellular calcium concentration, the intramembranous field is increased and the probability of channels being activated is decreased resulting in a reduction in excitability.

However, surface charge screening does not account for all of the calcium-dependent gating phenomena exhibited by voltage-gated channels. At its simplest the surface potential theory predicts a uniform action on all types of voltage-dependent channels. While sodium channel activation is enhanced by reductions in extracellular calcium other types of voltage-gate ion-channels exhibit different dependence on extracellular calcium, ranging from sensitive to indifferent (Han et al., 2015). Beyond charge screening, other work has identified at least two other distinct biophysical mechanisms through which changes in extracellular calcium can alter the activity of voltage-gated sodium channels (Armstrong and Cota, 1991). Voltage-gated sodium channels have a number of extracellular moieties that also interact directly with calcium and so alter channel kinetics through changes in conformation or stability (Armstrong and Cota, 1990). Also, calcium ions are able to directly block sodium channels likely through interactions with specific amino acid residues lining the channel's pore (Santarelli et al., 2007).

Direct activation of non-selective cation channels following reductions in extracellular calcium, also depolarizes neurons and increases excitability (Hablitz et al., 1986; Xiong et al., 1997; Immke and McCleskey, 2001). Calcium homeostasis modulator 1 (CALHM1) is another non-selective cation channel that is both voltage- and calcium-dependent (Ma et al., 2012) positioning it to be a strong mediator of calcium-dependent excitability. In fact, neurons deficient in CALHM1 lost all calcium-dependent excitability (Ma et al., 2012) implying that in some neurons surface charge screening may not contribute to calcium-dependent excitability.

### Receptor-mediated signaling of extracellular calcium

The excitability of neurons is also influenced indirectly by complex second messenger systems coupled to membrane receptors (Figure 1B). The calcium-sensing receptor (CaSR), a widely expressed G-protein coupled receptor, exhibits a punctate staining pattern in the cortex and cerebellum consistent with localization to nerve terminals (Ruat et al., 1995). Direct recordings from neocortical terminals, demonstrated that extracellular calcium regulated a membrane receptor and indirectly modulated a non-selective cation channel (Smith et al., 2004). Using a combination of pharmacological probes and a mutant mouse the terminal extracellular calcium receptor was identified as CaSR (Chen et al., 2010). In hippocampal neurons reductions in extracellular calcium increased neuronal excitability via another indirect mechanism. A non-selective cation channel (NSCC) NALCN (Na-Leak Channel Non-selective), was activated by decreases in extracellular calcium and mediated the vast majority of calcium-dependent excitability (Lu et al., 2010). This signaling pathway required two intracellular proteins, UNC-79 and UNC-80, and an unidentified membranous receptor (Lu et al., 2010). The authors went on to hypothesize that CaSR may be the receptor that detected and transduced changes in extracellular calcium into changes in neuronal excitability. In this model, low extracellular calcium was transduced into activation of a depolarizing current mediated by NALCN and increased neuronal excitability, controversially minimizing the contribution of surface charge screening (Lu et al., 2010). Other consequences of extracellular calcium signaling are suggested by work showing that CaSR may inhibit some neuronal potassium channels (Vysotskaya et al., 2014). Interestingly, CaSR activation was also proposed to activate other types of neuronal potassium channels (Vassilev et al., 1997a). Similarly, an unusually non-selective channel in neuronal soma was reported to be activated by CaSR agonists (Ye et al., 1996). The impact of decreased extracellular calcium on CaSR modulation seems to favor channel activation but the overall effect will depend on the balance of channel activation and block.

Intracellular changes in calcium, as a result of changes in extracellular calcium, may modulate channel activity and neuronal excitability. Calmodulin, a calcium sensitive signaling protein that is modulated by calcium entry, regulated sodium channel activity (Kim et al., 2004). Specifically, calmodulin interacted with an intracellular domain of voltage-gated sodium channels and so modified their gating behaviors (Sarhan et al., 2012). Notably, calmodulin has also been shown to regulate the cell surface expression and signaling from the CaSR providing a potential mechanism for cross-talk between these distinct calcium signaling pathways in the modulation of neuronal excitability (Huang et al., 2010). Thus, there are multiple direct and indirect mechanisms by which extracellular calcium can impact the intrinsic excitability of neurons and while surface charge theory provides a common mechanism across all neurons it would be surprising if these other mechanisms did not operate in parallel and mediate variability in calcium dependent excitability between neuronal types.

## Extracellular calcium and synaptic function

### Calcium is a key determinant of synaptic efficacy

Calcium is an important signal on both the pre- and post-synaptic sides of the synapse where it triggers exocytosis (Douglas, 1968; Katz, 1969), plasticity (Lynch et al., 1983; Malenka et al., 1988; Bliss and Collingridge, 1993) and alters gene expression (Greenberg et al., 1992). The early and reproducible observation that synaptic efficacy is dependent on the fourth power of extracellular calcium highlights the importance of calcium in the exocytotic process and has been confirmed in a number of preparations (Dodge and Rahamimoff, 1967; Dudel, 1981; Augustine and Charlton, 1986; Zucker et al., 1991; Bollmann et al., 2000; Schneggenburger and Neher, 2000). Calcium activates the exocytotic machinery after entry through N-, P/Q-, and R- type voltage-activated calcium channels (Wheeler et al., 1994; Jun et al., 1999; Wu et al., 1999; Rozov et al., 2001). Numerous forms of synaptic plasticity have been described with varied rates of onset and durations lasting from milliseconds to hours (Katz and Miledi, 1969; Lynch et al., 1983; Malenka et al., 1988; Bliss and Collingridge, 1993; Zucker, 1993; Fisher et al., 1997; DeMaria et al., 2001; Kreitzer and Regehr, 2001; Rozov et al., 2001), all of which are affected by cleft calcium emphasizing its important regulatory role on synaptic function.

### The impact of falls in cleft calcium

The broad dynamic range of extracellular calcium along with the exceedingly steep dependence of synaptic release probability on extracellular calcium (Dodge and Rahamimoff, 1967) leads to the hypothesis that even modest falls in cleft calcium will render the synapse much less effective at conducting signals. Indeed, a widely observed fourth-order proportionality implies that a reduction of the cleft calcium by one third could reduce synaptic efficacy by up to 80%. Accordingly, maneuvers reducing cleft calcium reduce synaptic efficacy (Borst and Sakmann, 1999a). Nevertheless, sustained phasic synaptic transmission has been observed at rates of up to 800 Hz (Taschenberger and von Gersdorff, 2000), indicating that either falls in cleft calcium do not occur at all synapses or there are compensatory mechanisms to reduce the effect of the fall of extracellular calcium at the synaptic cleft. The mechanism by which reductions in extracellular calciumreduce release probability and potential compensatory mechanisms remain incompletely understood, but similar to the impact of extracellular calcium on synaptic transmission can be divided into direct biophysical mechanisms and indirect mechanisms mediated by second messenger systems.

### Direct compensatory mechanisms

Dissociation of calcium from negative charged macromolecules, release from synaptic vesicles, and extracellular cation exchangers have been proposed to attenuate the fall in cleft calcium during episodes of high activity (Grohovaz et al., 1996; Borst and Sakmann, 1999a; Hartig et al., 2001), but the functional impact is uncertain. Similar to its impact on overall neuronal excitability, at the terminal reduced calcium is predicted to left-shift the voltage-dependence of sodium and calcium channels increasing the probability of release. Another putative, but incompletely understood compensatory mechanism observed at the calyx of Held and hippocampal nerve terminals is the broadening of presynaptic action potentials with repeated stimulation (Borst and Sakmann, 1999b; Geiger and Jonas, 2000). As calcium entry occurs during the repolarization phase of an action potential, spike broadening is a highly effective way of increasing calcium entry by prolonging depolarization (Sabatini and Regehr, 1997). Ion exchangers may also provide a mechanism to sustain synaptic transmission during periods of high activity. In parallel fiber-to-Purkinje neuron synapses, transient reversal of the sodium/calcium exchanger promotes calcium influx and enhanced glutamatergic transmission (Roome et al., 2013).

### Indirect compensatory mechanisms

There is considerable evidence that the CaSR is intimately involved with regulating synaptic transmission Figure 1C. The CaSR is present in 80–90% of nerve terminals in the cerebral cortex (Smith et al., 2004; Chen et al., 2010) and its impact on synaptic transmission is complex indicating that it may be mediated by several mechanisms. In acutely isolated neocortical nerve terminals, decreases in extracellular calcium activated voltage-dependent NSCC currents indirectly via the CaSR (Smith et al., 2004; Phillips et al., 2008; Chen et al., 2010). Theoretically, NSCC activation at the nerve terminal following decreased CaSR activation (Smith et al., 2004) may depolarize the local membrane potential, inactivate voltage-dependent calcium channels, and thereby reduce the probability of evoked release. However, the voltage-dependence of the terminal NSCC means very few of the NSCCs would be activated at negative potentials making this unlikely to be a major effect. Another possibility is that NSCC activity following reduced CaSR activation could lead to action potential broadening which might prolong the duration of calcium entry and facilitate synaptic transmission. The absence of delay of activation of NSCC currents following rapid depolarizations (sub millisecond) and the ability of action potential waveforms to trigger these currents supported this hypothesis (Smith et al., 2004). Consistent with this idea, CaSR activation reduced excitatory transmission between pairs of neocortical neurons (Phillips et al., 2008). Furthermore, deletion of CaSR substantially increased excitatory synaptic transmission in neocortical neurons, and variance-mean analysis indicated this was due to an increase in release probability (Phillips et al., 2008). Thus, CaSR-NSCC signaling in nerve terminals would seem ideally placed to serve to increase release probability in situations where extracellular calcium was low thereby maintaining the fidelity of synaptic transmission during periods of high activity. However, although the NSCC currents were rapidly activated and likely to influence action potential shape the CaSR is a GPCR and unlikely to respond rapidly. Indeed in isolated terminals the pathway took a few seconds to respond to changes in extracellular calcium. These relatively slow kinetics indicate the CaSR-NSCC signaling pathway in terminals is more likely to detect and respond to sustained changes in calcium that persist for a few seconds and not those that develop over a few milliseconds (Smith et al., 2004; Chen et al., 2010). Endogenous modulators of CaSR in the periphery include magnesium, L-amino acids, polyamines, and γ-glutamyl peptides besides calcium (Leach et al., 2015). It remains unclear how much these agents modulate signaling in neurons but identification of central actions may reveal other physiological roles for CaSR in neurons as suggested for beta-amyloid (Conley et al., 2009).

Increasing attention has turned to spontaneous release of neurotransmitters with the recognition that action potential-evoked and spontaneous release mechanisms are distinct (Kavalali, 2015). Interestingly, CaSR activation by direct and allosteric agonists stimulate release of glutamate independent of intracellular calcium (Vyleta and Smith, 2011). In addition, deletion of CaSR substantially reduced spontaneous glutamate release. In other words, CaSR activation had opposite effects on evoked and spontaneous release of the major excitatory neurotransmitter (Phillips et al., 2008; Vyleta and Smith, 2011). It is as yet unclear how CaSR could have opposite effects on exocytosis of these apparently distinct populations of vesicles that reside in the same nerve terminals. However, we recognize that these apparently opposite actions mechanistically mirror the actions that CaSR stimulation has on release of parathyroid hormone and calcitonin (Garrett et al., 1995). The importance of CaSR signaling at nerve terminals has also been emphasized by the finding that spontaneous release of GABA, the major inhibitory neurotransmitter, is also strongly enhanced by CaSR activation (Smith et al., 2012).

Given the apparent abundance of CaSR it seems surprising that a role for CaSR was not suggested sooner. However, CaSR signaling may have been difficult to detect because “physiological” experiments frequently employed supraphysiological levels of calcium and magnesium concentrations (Smith et al., 2004; Chen et al., 2010). This approach ensured CaSR was at near-saturation attenuated our ability to detect changes in CaSR signaling. Another confounder is that in studies of the effects of decreased extracellular calcium, magnesium concentrations were often increased with the presumption that magnesium would only obviate the effects of surface charge screening. Since CaSR and spontaneous release are stimulated by magnesium (Vyleta and Smith, 2011; Smith et al., 2012) this experimental approach minimized the contribution of CaSR. The importance of employing physiological concentrations of divalent ions was emphasized by comparing neuronal activity and synaptic transmission *in vivo* and in acute brain slices (Sanchez-Vives and McCormick, 2000; Lorteije et al., 2009).

## Clinical relevance in the nervous system

Over the past decade a number of reports have underlined the potential of CaSR as a therapeutic target in diseases of the nervous system. Familial idiopathic epilepsy was linked to dominantly inherited CaSR mutations across three generations (Kapoor et al., 2008). The signaling pathways by which changes in CaSR activity might relate to epilepsy are not known, but the evidence implicating the CaSR in neuronal excitability and maintenance of high-frequency synaptic transmission suggests a plausible mechanism by which changes in CaSR activity could underpin a disorder of neuronal activity. In parallel, CaSR levels have been found to be increased in animal models following induction of seizures as well as traumatic brain injury (Mudo et al., 2009; Kim et al., 2011) hinting at a potential role for CaSR in the development of epilepsy following status epilepticus or traumatic brain injury. Intriguingly, CaSR antagonists were shown to reduce CaSR expression levels, brain tissue loss and neurological deficits, in animal models of traumatic brain injury and cerebral ischemia (Kim et al., 2013, 2014). Furthermore, links between beta amyloid and CaSR signaling may be important in the development of Alzheimer's disease and hypoxic brain injury (Bai et al., 2015; Dal Pra et al., 2015).

## Conclusions

Extracellular calcium ions are recognized, like intracellular calcium ions, as important regulators of neuronal function in the central and peripheral nervous systems. The action of extracellular calcium is complex and its actions via CaSR and surface charge screening affect numerous ion channels impacting neuronal excitability and many forms of synaptic transmission. An important goal for the field is to determine the relative contributions of these signaling pathways to neuronal function to facilitate our understanding behind the role of CaSR signaling in pathogenesis of acute neurological diseases like stroke, traumatic brain injury, and epilepsy.

## Author contributions

All authors listed, have made substantial, direct and intellectual contribution to the work, and approved it for publication.

### Conflict of interest statement

The authors declare that the research was conducted in the absence of any commercial or financial relationships that could be construed as a potential conflict of interest.
